# Automated Algorithm for J-T_peak_ and T_peak_-T_end_ Assessment of Drug-Induced Proarrhythmia Risk

**DOI:** 10.1371/journal.pone.0166925

**Published:** 2016-12-30

**Authors:** Lars Johannesen, Jose Vicente, Meisam Hosseini, David G. Strauss

**Affiliations:** 1 Office of Clinical Pharmacology, Center for Drug Evaluation and Research, US Food and Drug Administration, Silver Spring, MD, United States of America; 2 BSICoS Group, Aragón Institute for Engineering Research (I3A), IIS Aragón, University of Zaragoza, Zaragoza, Spain; University at Buffalo–The State University of New York, UNITED STATES

## Abstract

**Background:**

Prolongation of the heart rate corrected QT (QTc) interval is a sensitive marker of torsade de pointes risk; however it is not specific as QTc prolonging drugs that block inward currents are often not associated with torsade. Recent work demonstrated that separate analysis of the heart rate corrected J-T_peak_c (J-T_peak_c) and T_peak_-T_end_ intervals can identify QTc prolonging drugs with inward current block and is being proposed as a part of a new cardiac safety paradigm for new drugs (the “CiPA” initiative).

**Methods:**

In this work, we describe an automated measurement methodology for assessment of the J-T_peak_c and T_peak_-T_end_ intervals using the vector magnitude lead. The automated measurement methodology was developed using data from one clinical trial and was evaluated using independent data from a second clinical trial.

**Results:**

Comparison between the automated and the prior semi-automated measurements shows that the automated algorithm reproduces the semi-automated measurements with a mean difference of single-deltas <1 ms and no difference in intra-time point variability (p for all > 0.39). In addition, the time-profile of the baseline and placebo-adjusted changes are within 1 ms for 63% of the time-points (86% within 2 ms). Importantly, the automated results lead to the same conclusions about the electrophysiological mechanisms of the studied drugs.

**Conclusions:**

We have developed an automated algorithm for assessment of J-T_peak_c and T_peak_-T_end_ intervals that can be applied in clinical drug trials. Under the CiPA initiative this ECG assessment would determine if there are unexpected ion channel effects in humans compared to preclinical studies. The algorithm is being released as open-source software.

**Trial Registration:**

NCT02308748 and NCT01873950

## Introduction

Delay of ventricular repolarization measured as prolongation of the heart rate corrected QT (QTc) interval, is used as a surrogate marker of increased risk for torsade de pointes, a potentially fatal ventricular arrhythmia [1, 2]. Most drugs that prolong the QTc interval block the human ether-à-go-go related (hERG) potassium channel [3]. However, not all drugs that block the hERG potassium channel and prolong the QTc interval are associated with a risk of torsade de pointes, likely due to the presence of additional inward current block (e.g. ranolazine and amiodarone) [4, 5]. The lack of specificity and increased focus on hERG potassium channel block and QTc prolongation has caused the discontinuation of new drugs, sometimes inappropriately [3] and has motivated the initiation of the Comprehensive *In vitro* Proarrhythmia Assay (CiPA) initiative [6, 7].

The overall goal of CiPA is to develop a new paradigm for cardiac safety evaluation of new drugs that provides a more accurate and comprehensive mechanistic-based assessment of proarrhythmia potential [6, 7]. Drugs will be assessed *in vitro* for their effects on up to seven human cardiac ionic currents, and the results will be integrated in an *in silico* model of the human ventricular cardiomyocyte to output a proarrhythmia risk score. The *in silico* results will be confirmed with *in vitro* assays of stem cell derived cardiomyocytes and with ECG assessments in Phase 1 clinical studies. In order to achieve this, it is necessary to establish which ECG biomarkers can be used to detect effects of specific cardiac ion channel block.

Recently, we published the results of a retrospective analysis of 34 drugs [8] and two prospective clinical trials [9, 10] showing that measurement of the heart rate corrected J-T_peak_ (J-T_peak_c) and T_peak_-T_end_ intervals can be used to differentiate between drugs that are selective hERG potassium channel blockers with high torsade de pointes risk from drugs that block the hERG potassium channel as well as additional inward currents (calcium or late sodium) and likely are associated with minimal-to-no risk for torsade de pointes risk. Subsequent comprehensive analysis in both clinical trials of eight ECG biomarkers for comparing J-T_peak_c, T_peak_-T_end_, QTc and five other T-wave morphology indices showed that J-T_peak_c is the most predictive biomarker of the presence of inward current block [11]. The J-T_peak_c interval could thus be used to confirm presence of inward current block as indicated the by CiPA preclinical assessment.

However, differences in the definition of the peak of the T-wave exist, which may be critical for defining the J-T_peak_c and T_peak_-T_end_ intervals. These differences are of particular importance in the presence of notched and flat T-waves [12, 13]. In addition, previous studies including the T_peak_-T_end_ interval have analyzed different ECG leads, most commonly V5 [13–15].

The goal of this paper is to describe a measurement methodology for the J-T_peak_c and T_peak_-T_end_ intervals that allows for consistent capturing of electrocardiographic biomarkers for assessing proarrhythmic risk, which could be included in Phase 1 studies to confirm results of a comprehensive preclinical assessment. In addition, a fully automated algorithm for measuring J-T_peak_ and T_peak_-T_end_ that is being released as open-source software is presented and compared to semi-automated measurements from the primary analysis of the ECGs in the clinical studies.

## Methods

### Data

ECGs included in this paper come from two previously conducted prospective clinical trials, which were approved by the FDA Research Involving Human Subjects Committee (RIHSC 13-011D and 14-022D) and the local IRB. Briefly, the two prospective clinical trials were crossover trials of 22 healthy volunteers. In the first study subjects received a single dose of a predominant hERG potassium channel blocker (dofetilide and quinidine), a multiple ion channel blocker (ranolazine and verapamil) and placebo (FDA study 1 [9]: NCT01873950). Subjects in the second study received multiple doses of a selective hERG potassium channel blocker (dofetilide and moxifloxacin) alone or in combination with a late sodium (mexiletine or lidocaine) or L-type calcium current blocker (diltiazem) and placebo (FDA study 2 [10]: NCT02308748). All subjects in both clinical trials gave written informed consent. Both studies included a 12-lead continuous ECG recording sampled at 500 Hz and an amplitude resolution of 2.5 μV, from which three 10 s ECGs were extracted at pre-defined time-points throughout each dosing period, with one time-point being before dose administration. The ECGs were extracted based on maximizing signal-to-noise ratio and stability of the heart rate [16]. For more details on study design, please see references 9 and 10 [9, 10].

Prior to semi-automated and automated ECG assessment, baseline wander was removed from the 10 s ECGs using cubic spline interpolation [17] and median beats were generated using the trigger points of the QRS detector [18]. Afterwards, the median beats were up sampled to 1 kHz using cubic spline interpolation.

### Semi-automated assessment strategy

Assessment of ECGs in both studies [9, 10] was carried out using a semi-automated measurement methodology. The first step was automated measurement of location of the first peak and end of the T-wave, determined using the tangent method in the vector magnitude lead computed using the Guldenring transform [19, 20]. Afterwards, the automatically determined fiducial points were reviewed by two independent ECG readers blinded to treatment and time using in-house developed software to help assist the ECG reader to identify onset, offset and peaks of ECG waveforms [18, 21]. If the reviewer determined it was necessary, the measurement was changed by seeding the automated algorithm on-screen or manual movement of the location. All ECGs from one subject were reviewed in one sitting to minimize intra-reader variability. Afterwards, if there was disagreement of the location of any fiducial point by 5 ms, presence of notch or whether the ECG was measureable, the ECG was re-reviewed blinded to previous annotation. Finally, if disagreement persisted during the second review an expert ECG reader was consulted to reconcile the measurements.

### Algorithm for automated measurement

The algorithm is divided into two parts, the first part identifies peaks and slurs relevant to determining the peak and offset of the T-wave and the second part revises the offset of the T-wave based on a set of offset candidates. The initial development of the algorithm was done using Matlab R2014b (Mathworks, Natick, MA, USA) and the final version of the algorithm was implemented in C++. The selection of features used by the algorithm was done performing a decision tree (J48 algorithm) analysis in the KNIME Analytics Platform 2.10.2 (KNIME.com AG, Zurich, Switzerland) [22].

**Fig 1:**
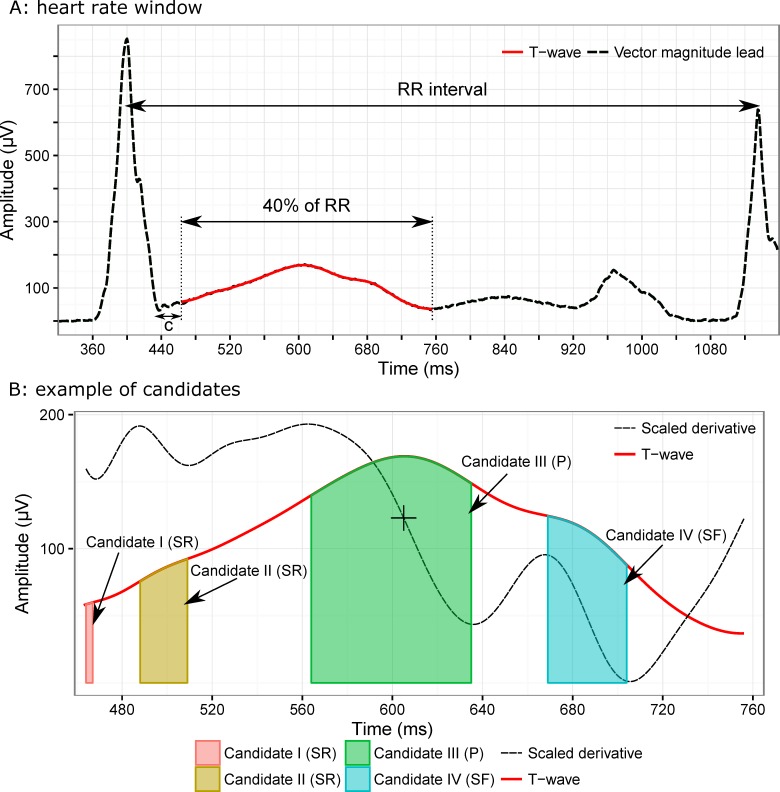
Graphical illustration of the T-wave delineation method Illustration of two steps in the T-wave delineation method described in the text. Panel A shows the definition of the empirical window based on heart rate where peak candidates will be detected. Panel B shows the smoothed derivative in solid black (scaled) together with the T-wave (red), and the four identified candidates: SR (slur–rising), peak and SF (slur–falling).

To identify peaks in the signal, a window of interest is automatically defined; this window begins 25 ms after the J-point (end of the QRS complex) and ends at 40% of the preceding RR (Fig 1A). The heart rate dependent window was designed empirically to help with performance during episodes of tachycardia. After defining the search window, all pairs of local maximum-minimum in the first derivative of a low-pass filtered T-wave are located as candidates (each candidate bounded between one local maximum and the following local minimum) (Fig 1B). Candidates are then either discarded based on their width (less than 10 ms to avoid small noise peaks), or identified as a peak or a slur based on the presence of a zero-crossing of the first derivative prior to the local minimum. If a candidate, which is wider than 10 ms, passes the zero line it is labeled as a peak, otherwise it is labeled as a slur. The peak of each candidate is the place of the highest amplitude of the candidate. The categorization of candidates into slurs and peaks is to help identify which slurs are relevant to determine the offset of the T-wave. Therefore, only the slurs following the peaks are kept, as they are likely most relevant to the identification of the offset. To identify the relevance of the slur for offset determination, a decision tree classifier was trained using a set of slur features identified based on feature extraction techniques (Table 1). After pruning the tree, three features (angle between two slopes (rising or left side and falling or right) of slurs; angle between peak and slur; and slur/peak amplitude ratio) were selected and implemented in the final version of the algorithm. To train the classifier, detected slurs of ECGs in FDA study 1 were tagged as being relevant for T-wave offset determination or not. Afterwards, the classifier was trained using 160 ECGs from FDA study 2, and the performance was evaluated using 240 test and 230 validation ECGs respectively, from the FDA study 2 and FDA study 1. The last step of the peak and slur identification process, was to apply a set of rules for final cleanup. These rules include merging candidates if the amplitude of the local minimum between two candidates is close to the amplitude of the peak of candidates, removing a peak with lower amplitude if the amplitude difference between two peaks was large, and converting a peak to a slur, if one side of the peak is almost horizontal. The peak of the T-wave is the highest peak between candidates at the final stage after applying the rules.

**Table 1:** List of features assessed during algorithm development.

Feature	Description
Y[p]/Y[s]	Amplitude ratio between peak and slur
Y[p]/Yorigin[s]	Amplitude ratio between peak and slur ^(^^a^^)^
Yorigin[p]/Yorigin[s]	Amplitude ratio between peak and slur
X[p]/X[s]	Time ratio between peak and slur
Y[p]-Y[s]	Amplitude distance between peak and slur
abs(Y[junctionX]-Y[p])	Amplitude distance between junctionX and peak
abs(Y[junctionX]-Y[s])	Amplitude distance between junctionX and slur
abs(Y[junctionX]/Y[p])	Amplitude ratio between junctionX and peak
abs(Y[junctionX]/Y[s])	Amplitude ratio between junctionX and slur
X[p]/junctionX	Time ratio between peak and junction
X[s]/junction	Time ratio between slur and junction
abs(X[p]-junctionX)/abs(X[s]-junctionX)	Time ratio between (peak-junctionX)/(slur-junctionX)
abs(Y[p]-Y[junctionX])/abs(Y[s]-Y[junctionX])	Amplitude ratio between (peak-junctionX)/(slur-junctionX)
(Y[p]-minVoltage)/divisor	Amplitude ratio between peak and divisor
(Y[s]-minVoltage)/divisor	Amplitude ratio between slur and divisor
abs(atand[p_rising_]–atand[p_falling_])	Angle between two slopes of peak (rising and falling slopes)
abs(atand[s_rising_]–atand[s_faling_])	Angle between two slopes of slur(rising and falling slopes) ^(^^a^^)^
abs(atand[p_falling_]–atand[s_rising_])	Angle between slopes of peak and slur(falling slope of peak and rising slope of slur) ^(^^a^^)^
atand(Rotation[p])	Angle rotation of peak
atand(Rotation[s])	Angle rotation of slur

p: peak; s: slur; X: time (ms); Y: maximum amplitude (μV); Yorigin: intersection between bisector two slopes(rising and falling) of a candidate and the vector magnitude lead signal; junctionX: intersection between rising slope of a candidate and falling slope of next candidate (similar to a local minimum between peak and slur); minVoltage: minimum voltage amplitude for detections (100 μV); divisor: abs(Y(junctionX)-minVoltage); atand: inverse tangent (degrees); Rotation: 90°-bisector angle between rising and falling slopes of a candidate (degrees)

^a^: selected features after pruning the decision tree and that were implemented in the C++ version of the algorithm (see C++ source code for more details).

The second step of the algorithm is to identify the offset of the T-wave. The offset of the T-wave was initially defined as the steepest slope based on the last identified candidate of the T-wave. However, during the development of the algorithm, extreme T-wave flattening was observed and the offset determination was changed as a result. The revised T-wave offset determination defines an interval of interest from the last candidate in the T-wave to the proposed T-wave offset (determined using steepest slope). In this interval, new candidates for the T-wave offset are identified using local minima of the energy signal. The energy is a cumulative amplitude signal calculated based on the first derivative that increases as the derivative goes up, but otherwise decreases. After, the energy signal is normalized the maximum value occurs at the beginning of the window and the lowest value at the end of the window. A new offset is chosen by minimizing a cost function that weighs: 1) the ratio between the distance of candidates to the T-wave peak and the distance of the T-wave peak to the next QRS complex and 2) the energy of candidates.

### Statistical analysis

The QT and J-T_peak_ intervals were computed relative to the semi-automatically identified QRS_onset_ and QRS_offset_ respectively, but using T_peak_ and T_end_ fiducial points automatically adjudicated by the algorithm using all ECGs from the two clinical trials. The measurements of the two methods (semi-automated vs. automated T_peak_ and T_end_) were compared using the methodology of comparing QT measurement algorithms proposed by Kligfield et al. for comparing the ability to detect moxifloxacin response of different QT measurement algorithms [23]. Briefly, the comparison consists of comparing the single-deltas using a Bland-Altman plot [24], evaluation of intra-time point variability and comparison of the mean and 95% confidence intervals. In addition, the slope of the exposure-response relationship was compared, as this is a critical component of evaluating drug-induced ECG effects during drug development [25]. The mean and 95% confidence intervals were computed using a linear mixed effects model with change from baseline as the dependent variable and treatment, time, sequence, period and an interaction between treatment and time as independent variables as well as a random effect on subject. The linear mixed effects model was fitted using lme4 and lsmeans in R 3.1.2 (R Foundation for Statistical Computing, Vienna, Austria).

## Results

### ECG examples

**Fig 2:**
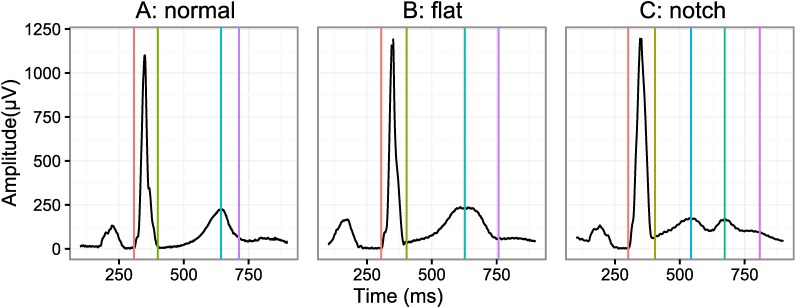
ECG examples. A set of ECG examples showing the different type of ECG morphologies observed in the two prospective clinical trials. From left to right the panels represent a normal T-wave (A), flattened T-wave (B) and a T-wave with a prominent notch (C), the latter is only observed in 0.8% of the ECGs in FDA study 1 and never observed in FDA study 2. Lastly, the vertical lines correspond to the QRS onset (red), QRS offset (green), primary T-wave (green), secondary T-wave (blue) and T-wave offset (purple) all as identified semi-automatically.

Example ECGs are shown in Fig 2, where each panel corresponds to a normal T-wave, flat T-wave, T-wave with a slur in the ascending limb and a notched T-wave (42 [0.8%] notched ECGs total in FDA study 1 and no notched T-waves in FDA study 2) for the vector magnitude lead.

### Evaluation of automated algorithm

**Fig 3:**
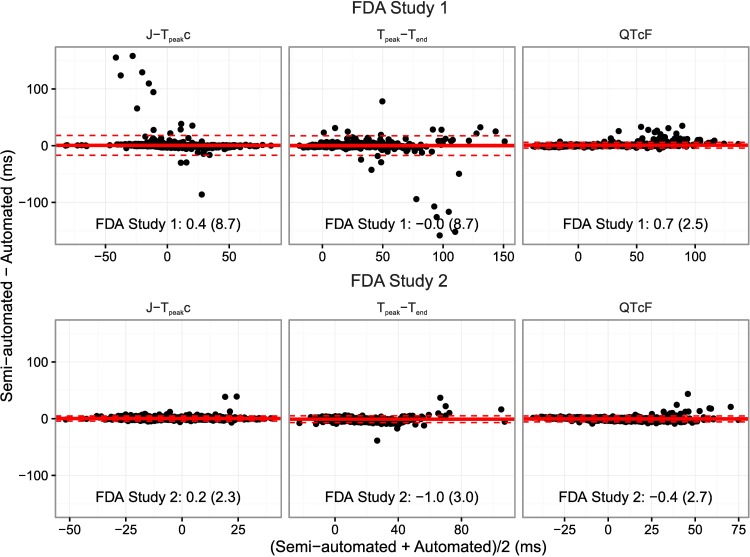
Bland-Altman plots. Bland-Altman plot for the J-T_peak_, T_peak_-T_end_ and QT intervals comparing semi-automated and fully automated measurements for FDA Study 1 [9] (top) and FDA Study 2 [10] (bottom). The solid horizontal lines represent the average difference and the dashed lines 2 times the standard deviation (SD) of the difference. In the bottom of each panel is the average difference (SD) of the single-deltas. Of note, only 8 and 9 measurements for J-T_peak_c and T_peak_-T_end_, respectively for FDA study 1, were associated with differences >40 ms due to borderline notches (10 single-deltas in total). Removal of these measurements results in a reduction of the SD to <3 ms for all intervals. A representative ECG from one of the eight single-delta outliers identified in Fig 3 is shown in Fig 4B. For cases like this, the blinded adjudicated interpretation was that the initial hump was not large enough to be considered a peak, however, the algorithm identified it a peak thus resulting in a large outlier (>40 ms). However, even in the presence of significant QTc prolongation (~80 ms) outliers of this type were rare (eight single-deltas). In addition, for FDA Study 1 there were a few single-deltas with a difference in QTc of ~30 ms, an example ECG is shown in Fig 4C, where the T-wave barely met the criteria for amplitude.

Fig 3 shows the Bland-Altman plots comparing all the single-deltas obtained by automated algorithm to the previously measured semi-automated measurements from the primary results for the two FDA studies. In FDA study 1, the single-deltas between automated and semi-automated measurements are comparable as is evident from the overall differences being within 1 ms and a SD of < 9 ms for both the J-T_peak_c and T_peak_-T_end_. Of note, if 10 single-delta outliers (out of 1635) associated with borderline notches are removed, the SD decreases to <3 ms. Moreover, the majority of the outliers are from two subjects near maximum concentration of dofetilide and quinidine that have extreme QTc prolongation (~80 ms) far beyond the normal level of regulatory concern (~10 ms). These results are comparable to the difference between two QT algorithms for detecting moxifloxacin effects, where a mean difference of <1 ms was observed with a SD of 9 ms [26]. Furthermore, in FDA study 2, which had less substantial QTc prolongation compared to FDA study 1, but still prolongation of ~40 ms, the average single-deltas difference were within 1 ms for all intervals with a SD of <3 ms.

**Fig 4:**
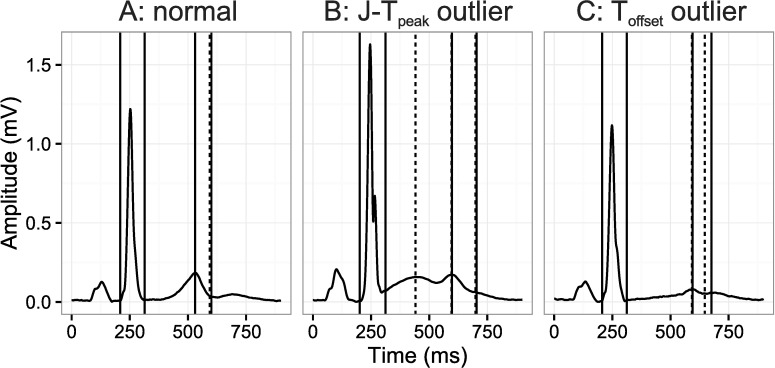
Example ECGs. An ECG with a small difference and two ECGs with outlier single-deltas (Fig 3). The left ECG (A) represents a normal ECG with small differences, middle ECG (B) representing an example with a large difference in the J-T_peak_ and T_peak_-T_end_ intervals and the right ECG (C) representing an example of a moderate difference (~30 ms) in QTc. The solid vertical lines refer to semi-automatic measurements and the dashed lines to fully automatic.

Both clinical studies had three replicate 10 ECGs at each time-point, which was used to assess the consistency of the measurements (intra-time point standard deviation), see Table 2. No differences in the intra-time point standard deviations were observed between semi-automated and fully automated measurements (p for all > 0.39, Table 2).

**Table 2:** Intra-time point standard deviation.

			Standard deviation of intra-time point measurements		
		N	J-T_peak_c	T_peak-_T_end_	QTc
FDA Study 1					
	Semi-automated	109	4.9 (4.6 to 5.1)	1.9 (1.6 to 2.2)	4.8 (4.5 to 5.1)
	Automated	109	5.0 (4.7 to 5.4)	2.0 (1.6 to 2.4)	4.7 (4.3 to 5.0)
	Difference	109	-0.2 (-0.6 to 0.3) p = 0.48	-0.1 (-0.6 to 0.4) p = 0.71	0.1 (-0.3 to 0.7) p = 0.63
FDA Study 2					
	Semi-automated	101	4.0 (3.7 to 4.2)	1.8 (1.5 to 2.1)	4.3 (3.9 to 4.6)
	Automated	101	3.9 (3.7 to 4.2)	1.6 (1.3 to 1.9)	4.1 (3.8 to 4.4)
	Difference	101	0.1 (-0.3 to 0.4) p = 0.74	0.2 (-0.2 to 0.6) p = 0.39	0.1 (-0.3 to 0.6) p = 0.53

Average (95% CI) of intra-time point SD by subject (averaged across periods). Comparisons between semi-automated and automated are performed using paired t-test.

**Fig 5:**
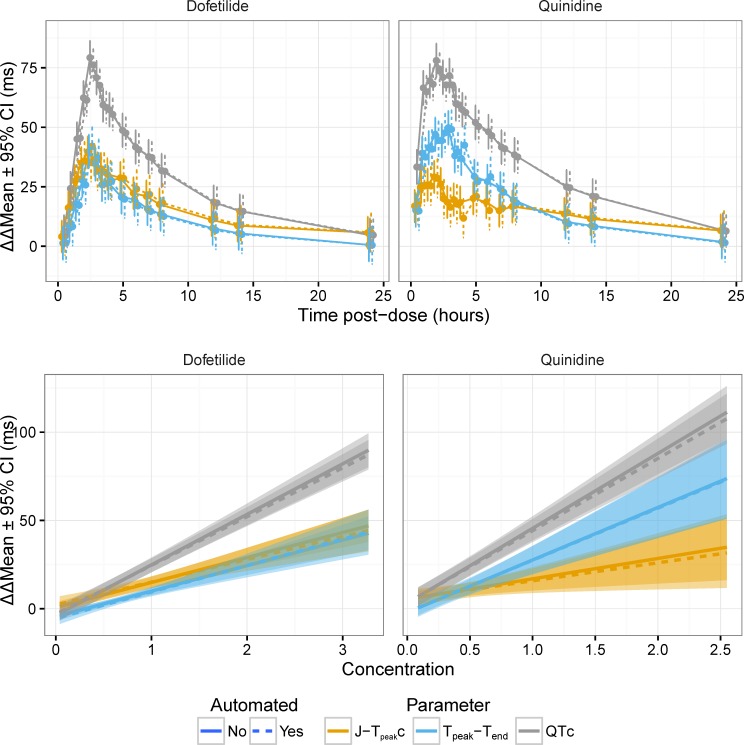
Time profiles and exposure-response relationship for dofetilide and quinidine. The plots in the top row show the time-profile for dofetilide (left) and quinidine (right) for J-T_peak_c (orange), T_peak_-T_end_ (blue) and QTc (gray) for semi-automated (solid lines) and automated (dashed lines). The plots in the bottom row show the corresponding exposure-response relationship.

**Fig 6:**
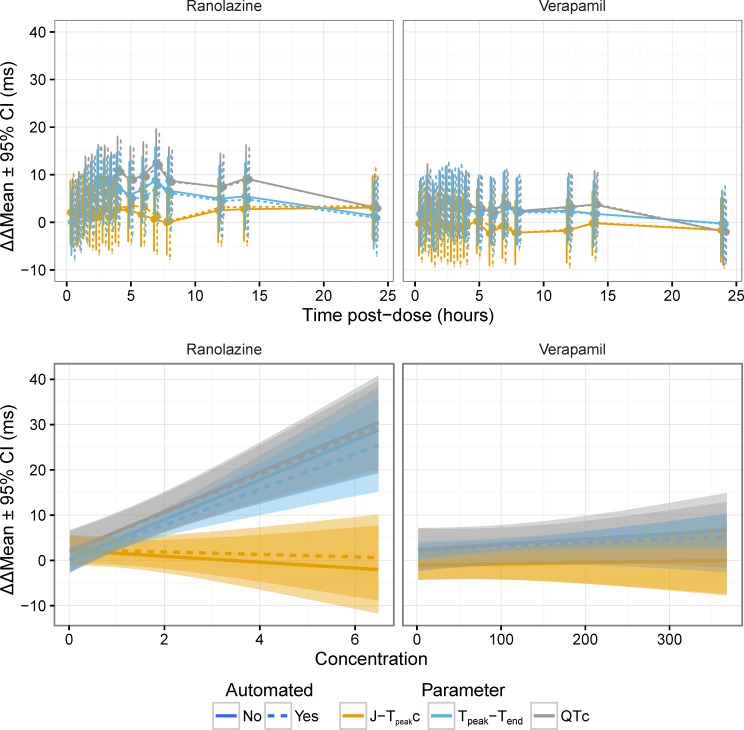
Time profiles and exposure-response relationship for ranolazine and verapamil. The plots in the top row show the time-profile for ranolazine (left) and verapamil (right) for J-T_peak_c (orange), T_peak_-T_end_ (blue) and QTc (gray) for semi-automated (solid lines) and automated (dashed lines). The plots in the bottom row show the corresponding exposure-response relationship. See. Fig 7 for time-profiles with some time points removed to enhance visualization due to crowding of early time-points.

**Fig 7:**
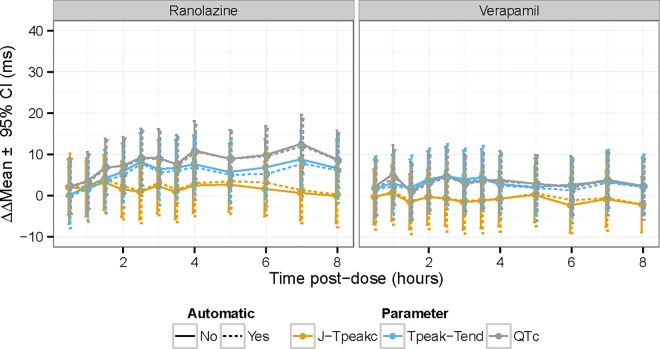
Time profile for ranolazine and verapamil with some time-points removed. The plots show the time-profile for ranolazine (left) and verapamil (right) for J-T_peak_c (orange), T_peak_-T_end_ (blue) and QTc (gray) for semi-automated (solid lines) and automated (dashed lines). This is the dame data as Fig 6 with some time points removed to enhance visualization due to crowding of early time-points.

**Fig 8:**
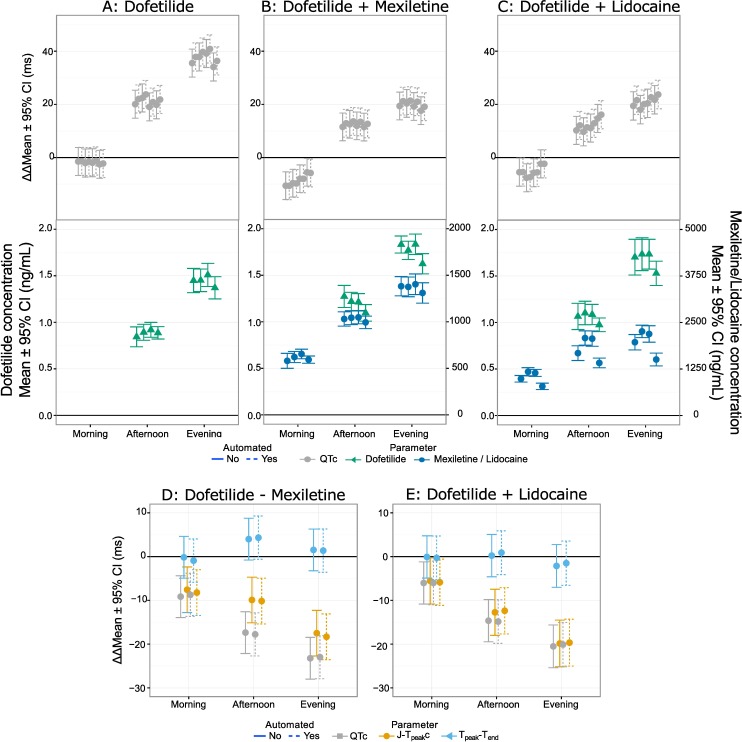
Time profiles for drug-combinations for FDA study 2 (primary). Time-profiles for the drug combinations studied in Johannesen et al. [10] are shown for QTc for dofetilide (A), dofetilide + mexiletine (B) and dofetilide + lidocaine (C) with corresponding time-profiles for the pharmacokinetic samples below. Panels D and E show the differences for QTc (gray), J-T_peak_c (orange) and T_peak_-T_end_ (blue) for dofetilide–mexiletine and dofetilide–lidocaine. In all the panels dashed are from automated measurements and solid are from semi-automated measurements.

**Fig 9:**
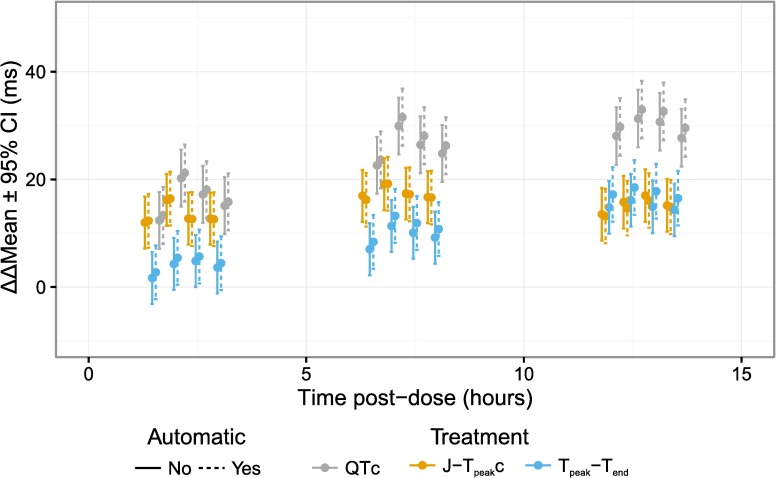
Time profiles for drug-combinations for FDA study 2 (secondary). Time-profiles for moxifloxacin with or without diltiazem for QTc (gray), J-T_peak_c (orange) and T_peak_-T_end_ (blue) from Johannesen et al. [10]. Dashed lines are from automated measurements and solid are from semi-automated measurements.

Most importantly, the time-profiles and exposure-response relationships were assessed for dofetilide and quinidine (Fig 5), ranolazine and verapamil (Fig 6 and Fig 7) and dofetilide with and without mexiletine or lidocaine (Fig 8) and moxifloxacin with and without diltiazem (Fig 9). As anticipated based on the comparison of the single-deltas there are almost no discernable differences in the time-profiles, with 63% of the time-points having less than 1 ms difference (86% within 2 ms) and all the confidence intervals overlap. These results are similar to numbers reported by Green et al. (7 out of 11 time-points) for comparison of QT algorithms to detect effects of moxifloxacin [26]. The exposure-response relationships obtained from automated and semi-automated measurements for dofetilide and quinidine (Fig 5) and ranolazine and verapamil exhibited overlapping population predictions (Fig 6). Importantly, the same conclusions for the ECG effects in both studies concerning relative prolongation of J-T_peak_c and T_peak_-T_end_ are drawn regardless of whether semi-automated or fully automated measurements are used.

## Discussion

This paper describes the details of a method for defining two ECG intervals (J-T_peak_ and T_peak_-T_end_). Through a retrospective analysis of 34 thorough QT studies and two prospective clinical trials [8–10], measurement of these intervals was determined to be critical for detecting the presence of inward current block (L-type calcium or late sodium), which provides insights into risk for torsade de pointes. These two ECG intervals were defined based on the measurements on the vector magnitude lead, and we have developed an automated algorithm that can measure these intervals, provided that the QRS_offset_ has been identified. The automated algorithm is capable of reproducing the time-profiles and exposure-response relationships of the semi-automated measurements for 8 drugs and 3 drug combinations in two prospective clinical trials and the algorithm is being released as open source software. Under a proposed new cardiac safety paradigm for assessing proarrhythmic risk of new drugs (the CiPA initiative), human Phase 1 ECG data would be used to determine if there are unexpected ion channel effects compared to preclinical ion channel data [6].

Prior studies demonstrated that the peak of the T-wave, as identified as the time point of the maximum value in the vector magnitude lead could be used to define two ECG subintervals: J-T_peak_c and T_peak_-T_end_ [8]. The rationale was that this would result in a global definition of early repolarization (J-T_peak_c), mainly reflecting the plateau of the action potential, and late repolarization (T_peak_-T_end_) reflecting the terminal part of repolarization. This definition is also supported by work by Fuller and colleagues who showed that the timing of the peak of the T-wave in the root-mean-square (RMS) lead, which is likely similar to the vector magnitude lead, corresponds to repolarization of 50% of the epicardial electrograms [27]. Similarly, recent findings by Meijborg et al. show that the peak of the T-wave marks the time of repolarization of 25% of the ventricular electrograms [28]. These findings suggest that the peak of the T-wave can be used to separate the repolarization interval of the ECG into J-T_peak_c (mainly due to changes in the plateau of the action potential) and T_peak_-T_end_. However, it is worth noting that the J-T_peak_c interval may be influenced by changes in the later part the action potential, as the peak is the location of the average action potential, and thus a subset of cells have repolarized. One alternative fiducial point that, at least theoretically, could be a more specific index for the plateau part of the action potential is the onset of the T-wave. However, the onset of the T-wave can be difficult to identify, especially in the presence of hERG potassium channel blocking drugs that lead to flat T-waves. Moreover, reproducible measurements of changes in the J-T_peak_c and T_peak_-T_end_ intervals using this definition of the peak have shown to provide value in differentiating selective hERG potassium channel block from multichannel block (hERG potassium + late sodium and/or calcium current block). An analysis of 34 drugs and a simulation study showed that the J-T_peak_c interval can be used to detect the presence of inward current block [8]. This is likely because of the impact of late sodium or L-type calcium current block is most prominent during the plateau phase of the action potential [29, 30]. These observations were confirmed in two prospective clinical trials, showing that the J-T_peak_c interval defined as described in this manuscript, is a marker of inward current block [9, 10].

There are different ways to define the peak of the T-wave, which is most important in the presence of substantial changes in the T-wave morphology such as flattened or notched T-waves. One definition of the peak of the T-wave for notched T-waves is the nadir between the two peaks. This definition was initially proposed by Emori and Antzelevitch based on observations in their canine wedge model [12]. Their findings in the wedge model suggested that the nadir of the T-wave coincides with the end of repolarization of the endocardium, and thus the distance between the nadir and the end of the T-wave (end of repolarization) could reflect transmural dispersion [12]. However, the link between the T-wave and transmural dispersion in whole human hearts is a subject of debate [31, 32]. An alternative definition for the peak of the T-wave is the maximum peak. This definition can result in large variability of the measurements, as the amplitude of the first and second deflection can be of similar amplitude and the smallest amount of notch can cause shifts between the first and second peak. Based on this we defined the peak of the T-wave as the first discernable peak in the T-wave, which is also similar to definition of the other waveforms in the ECG, e.g. the R-peak is the first positive peak in the QRS complex. This definition does have potential limitations as it becomes important to discriminate between slurs and peaks consistently (see Fig 4B). However, in both FDA clinical trials there was a high degree of agreement between two independent reviewers of the location of the peak (~98%), which demonstrates that it is possible to apply these criteria consistently [9, 10].

Interestingly, a recent simulation study conducted by Sadrieh and colleagues shows that in the presence of reduction of the hERG potassium channel current, that action potential durations of two distinct populations of cells could be identified corresponding to the two notches in the T-wave [33]. In addition, a recent *in vivo* study of dofetilide in dogs showed that dofetilide caused significant interventricular delay in repolarization and that for notched T-waves the two peaks could correspond to either ventricle repolarizing [34]. This observation is consistent with the *in silico* results presented by Sadrieh and colleagues and suggest that T-wave notches may arise as a result of increased spatial dispersion, which could potentially be due to a difference in expression of cardiac ion channels [35]. Fortunately, in the context of assessing drug-induced ECG changes in healthy volunteers this is a manageable problem, as only 42 notches were observed in the vector magnitude lead in FDA study 1 [9], which included two potent selective hERG potassium channel blockers (dofetilide and quinidine) causing T-wave notching and substantial QTc prolongation (~80 ms).

The definition of the peak of the T-wave both in the presence or absence of notches is further complicated by the choice of lead for the analysis. Prior studies evaluating changes in T_peak_-T_end_ have focused on the left precordial leads, primarily V5, because they were thought to provide an index of transmural dispersion of the left ventricular wall [13, 15]. Another study has used the RMS lead [36]. Rather than making measurements on a single lead, we used the vector magnitude lead to provide a global index of repolarization and reduce influence of changes in the cardiac axis [37]. The difference between the RMS lead and the vector magnitude lead is that the RMS lead considers leads I, II and V1-V6, while the vector magnitude lead is computed using the X, Y and Z leads, which are orthogonal. With normal ECG waveforms the two leads are likely similar. However, there may be subtle differences in the presence of notches that are confined to mid precordial leads. This is because the mid-precordial leads are assigned a low weight in the computation of the X, Y and Z leads [19], that form the vector magnitude lead, whereas the RMS lead weighs all the leads equally.

The differences between the automated and semi-automated single-delta measurements are <1 ms for all intervals with a standard deviation of the difference in most cases being <3 ms, comparable to differences between two QT algorithms to detect moxifloxacin [26]. In addition, the intra-time point variability was not different between the two algorithms. Importantly, the automated algorithm produced nearly identical time-profiles (63% of time-points within 1 ms and overlapping confidence intervals) and exposure-response relationships as the semi-automated measurements. All together, these results demonstrate that the automated algorithm is capable of reproducing the semi-automated measurements. In addition, the classification of peaks and slurs from the algorithm produces several diagnostics such as number of detected peaks and amplitude of peaks that are being used to develop metrics for determining which measurements needs to be reviewed by an ECG annotator.

### Limitations

While the results from two prospective clinical trials [9, 10] support that the J-T_peak_c interval can be used to detect the presence of inward current block, it is important to recognize that in the presence of extreme T-wave morphology changes the assessment of these intervals can be challenging. Data from FDA study 1 in particular suggest that obtaining reliable measurements in the presence of substantial T-wave morphology changes can be accomplished [9, 38]. However, notches in the vector magnitude lead were very infrequent in FDA study 1 (42 ECGs [0.8%] in total) and completely absent in FDA study 2.

Moreover, it should be noted that search windows on the ECG were defined using an empirically defined window based on heart rate. Such definitions may not hold up uniformly, and future research should consider defining the search window differently. This could be achieved by identifying the approximate location of relevant waveforms in the repolarization interval, and using this information to segment the repolarization interval into search windows, thus avoiding the need for an empirically defined search window. One potential strategy for approximating the location of waveforms could be based on decomposing the signal, e.g. using Gaussian-Mesa-Functions.[39] However, while the performance of the automated algorithm on the presented data sets is good, it is unknown whether the algorithm is reliable for ECGs with extreme tachycardia, which is the subject of continued work. Similarly, the performance of ECGs with poor signal quality has not been determined, however, it would seem appropriate for this application to exclude ECGs of poor signal quality.

## Conclusion

We have presented a definition for two ECG intervals (J-T_peak_c and T_peak_-T_end_) that allows for detection of the presence of inward current block, which is important for drug-induced torsade de pointes risk assessment. Moreover, we have developed a fully automated algorithm, which can reproduce the semi-automated measurements for 8 drugs and 3 drug combinations. The algorithm is being released as open source software to allow for widespread use and proposed to be used in clinical Phase 1 ECG analysis for new drugs under CiPA.
